# Mechanism of RPE Cell Death in α-Crystallin Deficient Mice: A Novel and Critical Role for MRP1-Mediated GSH Efflux

**DOI:** 10.1371/journal.pone.0033420

**Published:** 2012-03-19

**Authors:** Parameswaran G. Sreekumar, Christine Spee, Stephen J. Ryan, Susan P. C. Cole, Ram Kannan, David R. Hinton

**Affiliations:** 1 Arnold and Mabel Beckman Macular Research Center, Doheny Eye Institute, Los Angeles, California, United States of America; 2 Department of Ophthalmology, Keck School of Medicine of the University of Southern California, Los Angeles, California, United States of America; 3 Division of Cancer Biology and Genetics, Queen's University Cancer Research Institute, Kingston, Canada; 4 Department of Pathology, Keck School of Medicine of the University of Southern California, Los Angeles, California, United States of America; Roswell Park Cancer Institute, United States of America

## Abstract

Absence of α-crystallins (αA and αB) in retinal pigment epithelial (RPE) cells renders them susceptible to oxidant-induced cell death. We tested the hypothesis that the protective effect of α-crystallin is mediated by changes in cellular glutathione (GSH) and elucidated the mechanism of GSH efflux. In α-crystallin overexpressing cells resistant to cell death, cellular GSH was >2 fold higher than vector control cells and this increase was seen particularly in mitochondria. The high GSH levels associated with α-crystallin overexpression were due to increased GSH biosynthesis. On the other hand, cellular GSH was decreased by 50% in murine retina lacking αA or αB crystallin. Multiple multidrug resistance protein (MRP) family isoforms were expressed in RPE, among which MRP1 was the most abundant. MRP1 was localized to the plasma membrane and inhibition of MRP1 markedly decreased GSH efflux. MRP1-suppressed cells were resistant to cell death and contained elevated intracellular GSH and GSSG. Increased GSH in MRP1-supressed cells resulted from a higher conversion of GSSG to GSH by glutathione reductase. In contrast, GSH efflux was significantly higher in MRP1 overexpressing RPE cells which also contained lower levels of cellular GSH and GSSG. Oxidative stress further increased GSH efflux with a decrease in cellular GSH and rendered cells apoptosis-prone. In conclusion, our data reveal for the first time that 1) MRP1 mediates GSH and GSSG efflux in RPE cells; 2) MRP1 inhibition renders RPE cells resistant to oxidative stress-induced cell death while MRP1 overexpression makes them susceptible and 3) the antiapoptotic function of α-crystallin in oxidatively stressed cells is mediated in part by GSH and MRP1. Our findings suggest that MRP1 and α crystallin are potential therapeutic targets in pathological retinal degenerative disorders linked to oxidative stress.

## Introduction

Oxidative stress is a contributing factor to retinal pigment epithelial (RPE) cell dysfunction in age-related macular degeneration (AMD) [1], [2]. Characteristic features of early AMD include the accumulation of subretinal deposits between RPE and Bruch's membrane and RPE morphologic changes [1], [3]. Dysregulated growth factor expression, scavenger receptors, and the mTOR pathway have all been implicated in mediating or modulating these pathologic changes [4]–[8].

Redox of RPE also plays a critical role in combating oxidative stress [1]. Among the cellular antioxidant constituents, reduced glutathione (GSH) is the major non-protein thiol antioxidant with pluripotent functions [9], [10]. Even though GSH is synthesized in the cytosol, it is distributed in intracellular organelles such as endoplasmic reticulum, nucleus and mitochondria. GSH depletion has been attributed to apoptosis either by predisposing cells to apoptosis or by modulating mitochondrial membrane potential and subsequent activation of caspases [11]. Since mitochondrial GSH (mGSH) plays a significant role in cellular defense against pro-oxidants, depletion of mGSH poses a threat to cell viability.

Elucidating GSH transport mechanisms of different cellular compartments has received considerable recent attention. In the brain, release of GSH from astrocytes is an important component of GSH homeostasis [12]. Brain astrocytes maintain redox balance by the ATP-dependent extrusion of GSH by ATP-binding cassette transporter, multidrug resistance protein 1 (MRP1) [12]. Studies have demonstrated that both glutathione disulfide (GSSG) and GSH are substrates for MRP1 [12]–[15]. However, information on expression and regulation of proteins associated with GSH efflux in the retina is scarce [16]. Differences in mRNA expression of MRPs in different RPE cell lines was reported [17]. However, the role of efflux transporters, particularly MRP1 in GSH regulation in RPE cells under unstressed and stressed conditions has not been studied so far.

α-Crystallins have been found in many non-lenticular tissues including the retina [18]. αA and αB crystallin both serve a cell protection function and a chaperone function. In lens epithelial cells, α-crystallins are anti-apoptotic against UVA-irradiation and tumor necrosis factor-α stimulation [18], [19]. α-Crystallins also function as chaperones by preventing aggregation and pathologic protein misfolding [20]. Overexpression of either human HSP27 or αB crystallin resulted in increased total GSH levels and decreased basal levels of intracellular reactive oxygen species (ROS) [21], [22].

Our laboratory has investigated the role of α-crystallins in RPE cell physiology and their regulation by oxidative stress [23]. Lack of α-crystallins rendered RPE cells more susceptible to apoptosis caused by oxidative stress [23]. Overexpression of αA or αB crystallin had similar degrees of protection in lenticular as well as non-lenticular cells [24]. We showed that RPE cells lacking either αA or αB crystallin are equally susceptible to H_2_O_2_-induced oxidant insult [23]. Recently, we discovered that αB crystallin is secreted from RPE cells in exosomes, and exogenous αB crystallin protected RPE cells from oxidative stress-induced apoptosis [25].

The link between the protective function of α-crystallin and cellular antioxidant status is not well understood. Both GSH and redoxins are major factors with critical redox functions in RPE cells [26]. GSH levels are elevated in α-crystallin overexpressing human lens epithelial cells [27]. However, the nature and mechanism of GSH participation in the α-crystallin-mediated antiapoptotic function of RPE cells has not been studied. Here, we investigated the relationship between GSH, redoxins and the antiapoptotic function of α- crystallins in RPE. For the first time, we provide evidence that MRP1 plays a key role in maintaining cellular thiol homeostasis by regulating GSH efflux in RPE.

## Results

### α-crystallin overexpressing RPE cells are resistant to oxidative stress induced cell death

**Figure 1:**
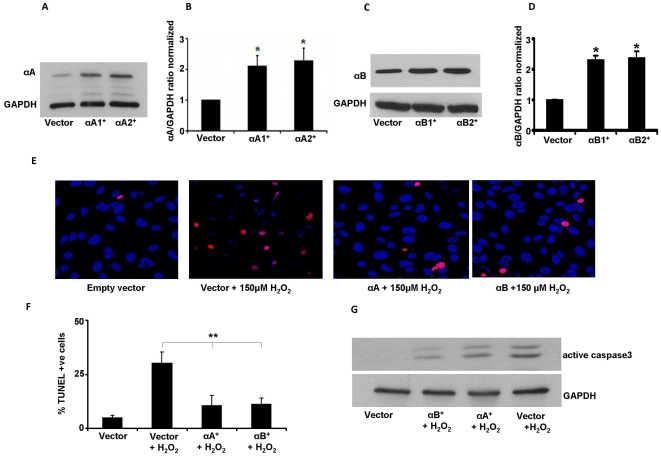
αA and αB crystallin protect RPE cells from H_2_O_2_-induced oxidative stress. αA and αB crystallin expression in two stably overexpressing ARPE-19 cell lines (αA1^+^, αA2^+^, αB1^+^, αB2^+^) was significantly higher than empty vector controls (A–D). Immunoblot analysis of ARPE-19 cells expressing αA crystallin and αB crystallin (A,C). Protein samples (25–75 µg) were separated by 15% Tris-HCL gel, transferred to PVDF membrane, and subsequently reacted with rabbit αA crystallin and αB crystallin antibodies. Protein expression quantified by densitometry is shown as ratio normalized with GAPDH (B, D). Cell death induced by H_2_O_2_ was significantly reduced in α-crystallin overexpressing ARPE-19 cells (E). α-Crystallin overexpressing cells were incubated with 150 µM H_2_O_2_ for 24 h in serum free medium and cell death was analyzed by TUNEL staining and semi-quantification of TUNEL positive cells are presented as percentage of dead cells in F. Blue: DAPI nuclear staining; Red: TUNEL positive cells. Caspase 3 activation with H_2_O_2_ in α-crystallin overexpressing cells (G). Twenty four hours after treatment, cell lysates (60 µg total protein) were run in inmmunoblots for active caspase 3 and then reprobed for GAPDH as a loading control. αA^+^ -αA crystallin overexpressing clones, αB^+^ -αB crystallin overexpressing clones, αA- αA crystallin, αB- αB crystallin. ** P<0.01 H_2_O_2_ vs αA^+^ and αB^+^. * P<0.05 Vector vs α-crystallin clones.

We generated α-crystallin overexpressing stable cell lines (Fig. 1 A–D) and demonstrated that αA crystallin or αB crystallin overexpressing cells were more resistant to H_2_O_2_-induced cell death than vector control cells (Fig. 1E). Overexpression of αA crystallin or αB crystallin resulted in 10% cell death at concentrations of H_2_O_2_ that caused 30% cell death in control cells (Fig. 1E, F). Further, caspase 3 activation was inhibited in α-crystallin overexpressing cells exposed to H_2_O_2_ (Fig. 1G). The dose and duration of H_2_O_2_ used in these studies were 150 µM and 24 h, respectively, as has been validated in our previous work [23].

### Higher thiol levels provide protection from oxidative stress in α-crystallin overexpressing cells

**Figure 2:**
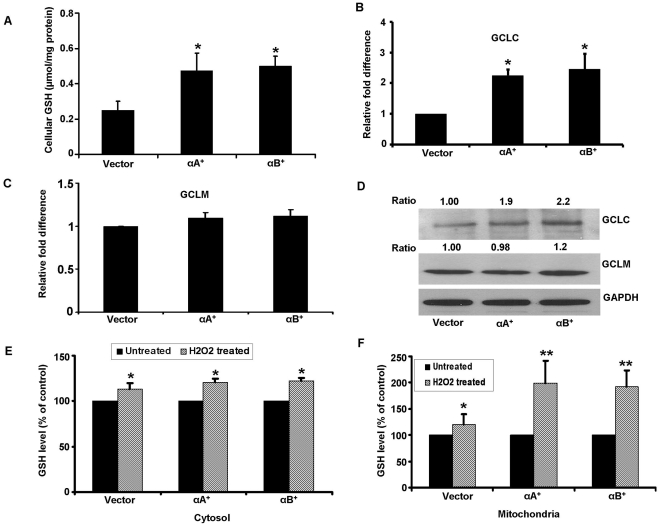
Intracellular GSH levels in α-crystallin overexpressing cells. Cellular GSH levels in ARPE-19 cells stably overexpressing αA crystallin and αB crystallin increased significantly (P<0.05) when compared to vector only cells (A). Relative expression of the mRNA levels of the catalytic unit (GCLC) and modifier unit (GCLM) of GSH biosynthetic enzyme GCL (B, C). A significant increase in GCLC was found for mRNA and protein while no significant change was observed either at the gene or protein levels for the modifier unit (B–D). Protein bands were quantified and presented as ratio normalized to loading control, GAPDH. Panels E and F show GSH levels in the cytosol and mitochondrial fractions from vector control and α-crystallin overexpressing ARPE-19 cells challenged with 150 µM H_2_O_2_ for 24 h. While the magnitude of increase in the cytosolic pool is relatively smaller, a significant (P<0.01) increase in mitochondrial pool of GSH was observed in α-crystallin overexpressing cells. Data are normalized to control taken as 100%. *P<0.05 vs controls; ** P<0.01 vs controls.

We next investigated the link between α-crystallin expression, intracellular thiol levels and enhanced cell survival in oxidative stress. Our data revealed a significant (P<0.05) 2-fold increase in cellular GSH levels in α-crystallin overexpressing clones when compared to controls (Fig. 2A). One of the main mechanisms for elevation of cellular GSH is increased biosynthesis catalyzed by the rate-limiting enzyme glutamate-cysteine ligase (GCL) [27]. The increase in total GSH levels was associated with significant upregulation of the gene and protein expression of the catalytic unit of GCL (GCLC) but not GCLM, the modifier unit of GCL (Fig. 2B–D). Mitochondrial fractions from α-crystallin overexpressing cells had significantly higher GSH levels (P<0.01 vs vector control) after treatment with 150 µM H_2_O_2_ for 24 h (Fig. 2E, F). The magnitude of increase in GSH level in cytosol, although higher than controls, was less than that of the mitochondrial fraction. Overall, these results suggest the significance of GSH and its biosynthetic enzymes in protection against oxidant stress in ARPE-19 cells overexpressing α-crystallins.

### Thiol status of the retina is compromised in α-crystallin KO retina

**Figure 3:**
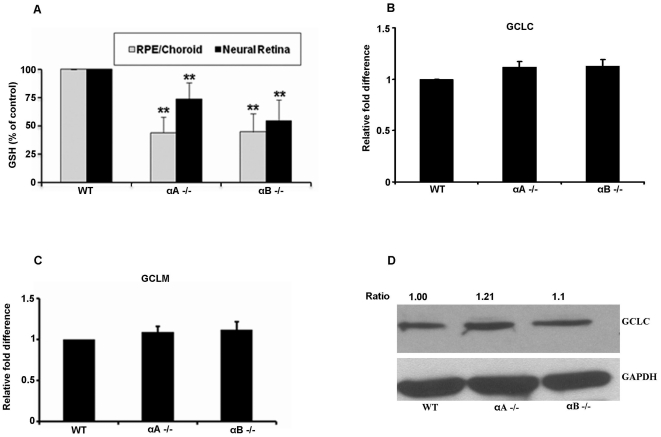
Cellular GSH levels in the αA and αB crystallin knockout (KO) mouse retina. (A) GSH levels in two tissue segments of the eye, namely RPE/Choroid and neural retina. Data, presented as percentage over control age-matched mice, showed a significant 50% decrease in GSH level in the neural retina and a ∼25–30% decrease in the RPE/choroid in αA crystallin KO (αA −/−) and αB crystallin KO (αB −/−) samples. RNA and protein were extracted from the posterior eye cup of the crystallin KO and WT mice. The gene expression of the catalytic (B) and the modifier unit (C) as well as the protein expression (D) of the GSH rate limiting enzyme GCLC did not show any significant change among αA (−/−), αB(−/−) and WT mice. GCLC- glutamate-cysteine ligase, catalytic subunit, GCLM- glutamate-cysteine ligase, modifier subunit, KO- knock out, WT- wild type. ** P<0.01 vs controls.

Retinas from α-crystallin KO mice are highly susceptible to cobalt chloride-induced oxidative stress [28]. We studied the changes in thiol status in mouse retina lacking αA-or αB crystallin. Total GSH levels in the neural retina and choroid/RPE complex of the α-crystallin KO and WT controls were determined. Under unstressed conditions, GSH levels were about 50% lower (P<0.01 vs age-matched WT) in αA crystallin and αB crystallin KO RPE/choroid complex while corresponding neural retina showed a 30% and 50% decrease in GSH, respectively (Fig. 3A). No significant changes in the levels of the catalytic or modifier subunits of GCL in αA and αB crystallin KO mice were found either at the mRNA (Fig. 3B, C) or the protein level (Fig. 3D) of samples from the posterior eye cups (neural retina, RPE and choroid). Additionally, we determined the effect of α-crystallin KO on the expression of thioredoxins (Trx) and glutaredoxins (Grx) in the retina and in the RPE cells [26]. Trx1, Trx2, Grx1 and Grx2 mRNAs were significantly (P<0.01) downregulated in α-crystallin KO retina (Fig. S1A). Similarly, Trx1, Trx2, and Grx1 protein levels were also downregulated in αA crystallin KO retina (Fig. S1B).

### GSH efflux in α-crystallin KO and α-crystallin overexpressing cells

**Figure 4:**
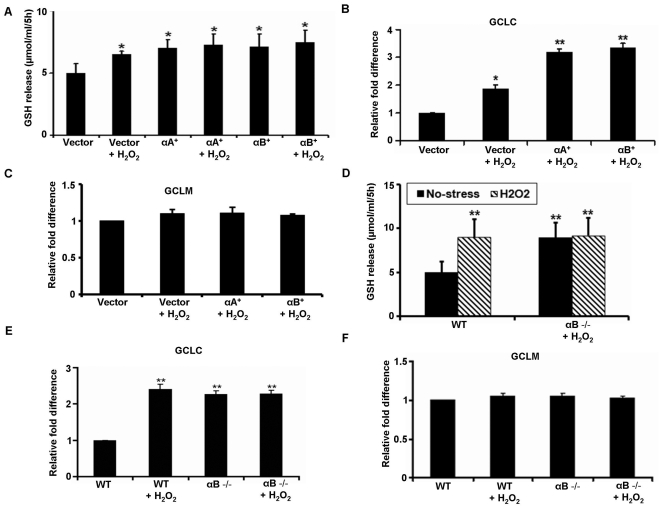
GSH export from H_2_O_2_-treated α-crystallin overexpressing and αB crystallin KO RPE cells. αA crystallin and αB crystallin overexpressing cells were treated with 150 µM H_2_O_2_ for 5 h in serum-free medium and extracellular accumulation of GSH was measured. Vector only cells treated in the same fashion served as controls (A). A significant (P<0.01 vs control) gene upregulation of GCLC (B) was observed while no significant change occurred in GCLM (C). GSH efflux in non-stressed RPE cells isolated from αB crystallin KO mice showed a significant increase when compared to RPE cells isolated from WT mice (D). However, oxidative stress (150 µM H_2_O_2_ for 5 h) resulted in a significant (P<0.01) increase in GSH efflux only in the WT RPE and no further significant increase in efflux was observed in RPE from αB crystallin KO mice. H_2_O_2_-induced stress produced a significant upregulation of the GCLC (E) while GCLM did not show any significant change (F). GCLC- glutamate-cysteine ligase, catalytic subunit, GCLM- glutamate-cysteine ligase, modifier subunit, WT- wild type. *P<0.05, ** P<0.01.

A major determinant of intracellular GSH levels is GSH efflux [29]. GSH efflux was significantly (P<0.01) higher in α-crystallin overexpressing cells when compared to vector control cells (Fig. 4A). Exposure to H_2_O_2_ did not further increase the amount of GSH released from α-crystallin overexpressing cells; however, GSH release was significantly (P<0.05) increased in H_2_O_2_-treated vector control cells (Fig. 4A). A significant upregulation of GCLC was observed in the α-crystallin overexpressing cells with H_2_O_2_ with no apparent change of the GCLM (Fig. 4B, C). On the other hand, in αB crystallin KO RPE cells, unstimulated GSH efflux amounted to 9 µmol/ml in 5 h which was significantly (P<0.05) higher than the 5 µmol/ml in 5 h in WT RPE cells (Fig. 4D). A significant increase in GSH release was found when WT RPE cells were challenged with 150 µM H_2_O_2_ for 5 h (Fig. 4D). This increase in GSH release could be attributed to an increase in GSH biosynthesis since GCLC levels were significantly higher (Fig. 4E) in RPE isolated from αB crystallin KO mice. However, no further increase in GSH efflux was seen in αB crystallin KO RPE exposed to the same concentration of H_2_O_2_ (Fig. 4F).

### MRP-related GSH transporters in RPE cells

We then proceeded to characterize the transporter(s) mediating GSH (and GSSG) efflux from RPE cells. Several MRPs are known to mediate GSH efflux in mammalian cells [9], [30]. To determine the presence of MRPs in RPE, MRP mRNA levels were analyzed by RT-PCR. RNA isolated from RPE cells was amplified using specific MRP primer sequences (Table S1). mRNAs encoding for MRP1, MRP2, MRP3, MRP4, MRP5, MRP6, and MRP7 were detected in RPE cells (Fig. S2A). MRP1 was the most abundant of the MRP family members in RPE (Fig. S2B). All further experiments were performed with MRP1 because it is the most well characterized MRP with respect to efflux of GSH and GSSG [30].

### Localization of MRP1 in α-Crystallin overexpressing RPE cells

**Figure 5:**
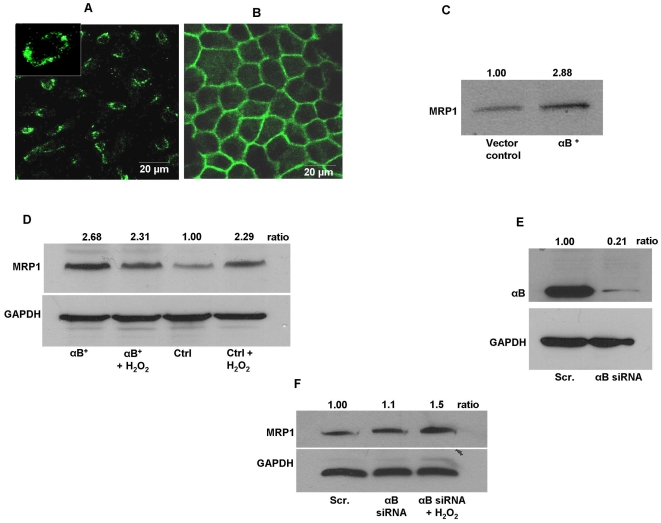
MRP1 is localized to the plasma membrane in human RPE cells. ARPE-19 cells (A) and polarized RPE monolayer from fetal human RPE cells (B) were fixed and incubated with a monoclonal antibody against MRP1 followed by a fluorescence labeled anti-mouse secondary antibody. Images were taken on a confocal laser-scanning microscope. (C) Vector control and αB crystallin overexpressing cells were labeled with biotin and surface labeled protein samples were analyzed by immunoblot for MRP1. A significant almost 3 fold increase in MRP1 protein expression was found. (D) MRP1 protein expression in vector control and αB crystallin overexpressing cells with and without oxidative stress. Cells were incubated with 300 µM H_2_O_2_ for 36 h in serum-free medium and total cell lysate was subjected to immunoblot analysis for MRP1 protein. Expression of MRP1 protein was >2 fold in αB crystallin overexpressing cells as compared to vector control cells. Treatment with H_2_O_2_ did not significantly alter MRP1 expression in αB crystallin overexpressing cells, however, a significant >2-fold increase was observed in vector control cells exposed to H_2_O_2_. (E) ARPE-19 cells were transiently transfected with scrambled and αB crystallin siRNA (50 nM) and protein was harvested 72 h post transfection. Whole cell lysates (20 µg of total protein) were subjected to immunoblot analysis using a rabbit polyclonal antibody against αB crystallin. αB crystallin protein expression was markedly reduced by >80% in siRNA transfected cells 72 h post-transfection. (F) MRP1 expression in αB crystallin-silenced cells with and without oxidative stress with H_2_O_2_. Transfected cells were treated with 200 µM H_2_O_2_ for 24 h in serum free medium and total protein (100 µg) was subjected to immunoblot analysis. While there is no apparent change in MRP1 protein expression in αB crystallin-silenced cells, H_2_O_2_ increased MRP1 expression 1.5 fold in αB crystallin-silenced cells. GAPDH was used as a loading control for all immunoblot analyses. GAPDH- Glyceraldehyde 3-phosphate dehydrogenase, MRP1- Multidrug resistance protein 1, αB^+^- αB crystallin. Scr. -Scrambled.

In subconfluent ARPE cells, MRP1 is predominantly localized in the plasma membrane and the staining pattern is punctate (Fig. 5A). In human polarized RPE monolayers, we observed lateral membrane localization of MRP1 (Fig. 5B). Biotinylation of intact cells with subsequent immunoblot analysis revealed surface localization of MRP1 in the membrane fraction (Fig. 5C). These studies further established that membrane expression of MRP1 was almost three fold higher (P<0.01) in αB crystallin overexpressing cells than vector control cells (Fig. 5C) which correlated well with the increased GSH efflux in α-crystallin overexpressing cells (*cf* Fig. 4A). In addition, cellular MRP1 expression showed a >2.5 fold increase in αB crystallin overexpressing cells (Fig. 5D) as compared to vector control cells. Furthermore, consistent with GSH efflux under oxidative stress (300 µM H_2_O_2_ for 36 h), we observed a >2 fold increase in MRP1 expression only in vector control cells subjected to oxidative stress.

Having established that increased α-crystallin levels increased MRP1 expression, we then investigated whether knocking down of αB crystallin could affect the expression of MRP1. As seen in Fig. 5E, a significant (P<0.001) suppression of αB crystallin was achieved by siRNA silencing. MRP1 expression tended to increase in αB crystallin knock-down RPE cells and treatment with H_2_O_2_ (200 µM for 24 h) further increased MRP1 expression by 1.5 fold (Fig. 5F). These studies suggest that MRP1 regulates GSH efflux under conditions of oxidative stress in RPE cells.

### Effect of MRP1 inhibition on GSH release and cell death

**Figure 6:**
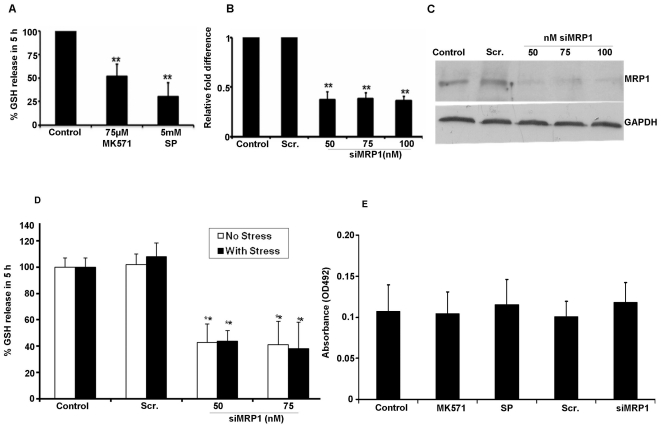
Inhibition of MRP1 significantly decreases basal and apoptotic GSH efflux. GSH efflux in ARPE-19 cells incubated with two MRP1 inhibitors (75 µM MK571 and 5 mM sulfinpyrazone [SP]) for 5 h (A). ARPE-19 cells were transfected with MRP1 siRNA at the indicated concentrations. RNA and protein were extracted 48 h post transfection. MRP1 mRNA and protein expression was significantly (P<0.01 vs scrambled control) decreased when compared to control or scrambled siRNA alone (B,C). GSH release from MRP1-silenced ARPE-19 cells incubated with 150 µM H_2_O_2_ for 5 h in serum free medium. A significant decrease (P<0.001 vs scrambled control) in GSH release was observed in 5 h. A decrease in GSH release only in control cells with no additional change in MRP1 inhibited cells was found and H_2_O_2_ treatment did not cause any further change (D). LDH release measured in MRP1 inhibited cells was not affected by any treatment (E). Values are mean± SE (n = 3–4). LDH- Lactate dehydrogenase. ** Significantly different from control cells, P<0.01.

To further confirm that MRP1 is involved in GSH efflux in RPE cells, we inhibited MRP1 by pharmacological agents and siRNA-mediated gene silencing. When serum-starved RPE cells were treated with MRP inhibitors (75 µM-MK571 or 5 mM-sulfinpyrazone) for 5 h, a significant 50% (P<0.01) decrease in GSH efflux was observed (Fig. 6A). MK571 and sulfinpyrazone are non-specific MRP inhibitors and can therefore be expected to inhibit some or all the MRP isoforms present in RPE cells (Fig. S2A).

To delineate the specific role of MRP1 in GSH transport, we selectively suppressed its expression by RNA interference. ARPE-19 cells were transfected with MRP1-specific siRNA duplexes and after 48 h, mRNA and protein levels of MRP1 were reduced by approximately 65% (Fig. 6B) and 70–80% (Fig. 6C), respectively. MRP1 silencing caused a significant 60% reduction (P<0.01 vs scrambled control) in GSH efflux from unstressed cells in 5 h (Fig. 6D). No further change in GSH efflux was observed in oxidatively stressed cells after incubation with 150 µM H_2_O_2_ for 5 h (Fig. 6D). Incubation of RPE cultures with MRP1 inhibitors or by MRP1 siRNA at the indicated doses and duration did not affect cell viability (Fig. 6E).

**Figure 7:**
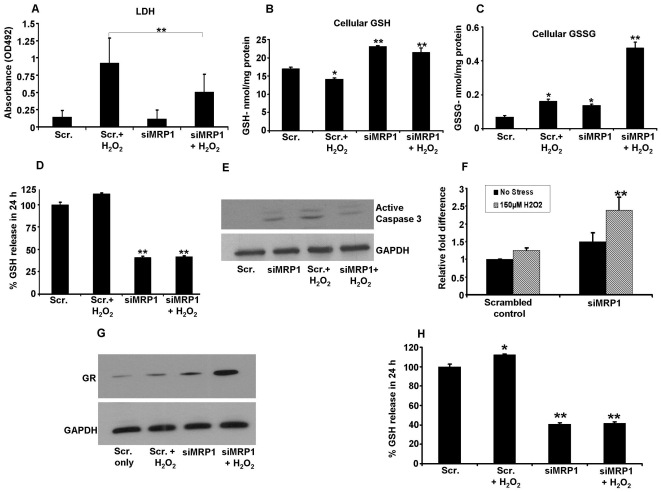
MRP1-inhibited RPE cells are resistant to cell death. MRP1 silenced and control cells were incubated with 150 µM H_2_O_2_ for 24 h in serum-free medium. 24 h LDH release was quantified in a 96 well plate reader. LDH release was significantly reduced in MRP1 silenced cells challenged with H_2_O_2_ when compared to similarly treated scrambled transfected cells (A). Cellular GSH (B) and GSSG (C) levels were significantly higher in MRP1 silenced cells when compared to scrambled controls. GSH efflux was inhibited by 60% in MRP1 silenced cells and did not change further with oxidant injury (D). Caspase activation, determined by immunoblot of active caspase 3 was reduced in MRP1-silenced cells challenged with H_2_O_2_ when compared to scrambled control cells (E). Glutathione reductase (GR) was significantly upregulated at the mRNA (F) and protein levels (G) in MRP1 silenced cells exposed to H_2_O_2_. Densitometric values are presented as ratio normalized to control (H). * P<0.05, ** P<0.01.

MRP1 down regulated cells were resistant to H_2_O_2_-induced (150 µM for 24 h) cell death (Fig. 7A). Cellular GSH levels in the scrambled siRNA and MRP1 siRNA groups were 16.99±0.45 and 23.11±0.20 nmol/mg cellular protein, respectively. However, there was a significant decrease (P<0.05) in GSH levels in control cells treated with H_2_O_2_ and no further change of GSH levels in MRP1 silenced cells (Fig. 7B). Cellular GSSG levels increased significantly (P<0.05) in control cells treated with H_2_O_2_ (Fig. 7C). In MRP1 silenced cells, the basal level of GSSG was higher than control cells and when incubated with H_2_O_2_, a 6.8 fold increase in GSSG levels was observed over scrambled controls (Fig. 7C). GSH efflux decreased by a significant 60% in MRP1 silenced cells with no further change with H_2_O_2_ treatment (Fig. 7D). The corresponding efflux for GSSG in MRP1 silenced cells was negligible and was below the detection limit of the assay (data not shown). Exposure of siRNA-MRP1 treated cells to H_2_O_2_ for 24 h led to a significant decrease in active caspase 3 levels when compared with corresponding control cells treated with H_2_O_2_ (Fig. 7E). Reduced cell death in MRP1 silenced cells treated with H_2_O_2_ under conditions of higher cellular GSSG may in part be due to increased glutathione reductase (GR) resulting in increased conversion of GSSG to GSH (Fig. 7F–H). Overall, these data support the conclusion that inhibition of MRP1 protects RPE cells from H_2_O_2_-induced cell death which is mediated by changes in thiol status and GR.

### Increased GSH efflux and susceptibility to cell death in MRP1 overexpressing cells

**Figure 8:**
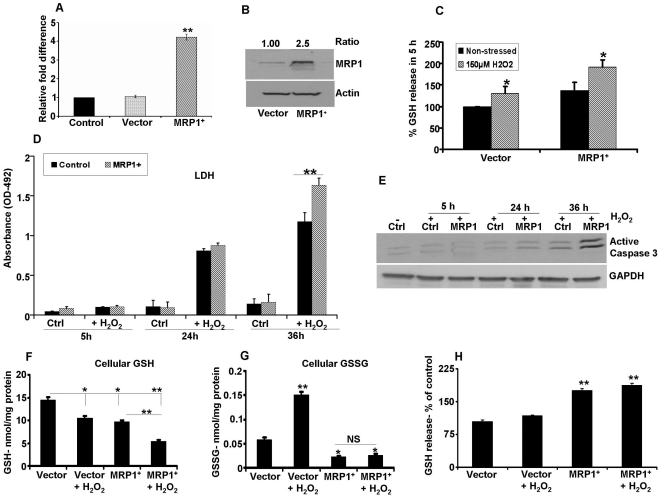
GSH efflux in MRP1 overexpressing ARPE-19 cells. (A) Real-time PCR was performed from RNA extracted from control (parental cells), vector transfected cells and cells transfected with human MRP1. MRP1 mRNA levels were normalized to GAPDH and data are presented as relative fold change over control cells. (B) Immunoblot analysis for MRP1 protein expression. A significant 2.5 fold increase in MRP1 was found. (C) MRP1 overexpressing cells release significantly higher quantities of GSH when compared to control cells. Oxidative stress (150 µM H_2_O_2_ for 5 h) further increased GSH release from control as well as from MRP1 overexpressing cells. (D) LDH release was not significantly altered in cells challenged with H_2_O_2_ for 5 h in both control and in MRP1 overexpressing cells. However, LDH release showed a progressive increase when H_2_O_2_ exposure was extended to 24 h and 36 h (n = 9). (E) A time dependent activation of caspase 3 expression was found with H_2_O_2_ treatment and this increase was maximal at 36 h. Cellular GSH (F) and GSSG (G) levels were significantly decreased in MRP1 overexpressing cells and oxidative stress with H_2_O_2_ further decreased GSH levels. GSSG levels showed a significant increase in H_2_O_2_ treated vector control cells and were very low in MRP1 overexpressed cells in the presence or absence of H_2_O_2_. (H) GSH efflux was higher in MRP1 overexpressed cells and oxidative stress further increased this efflux. * P<0.05, ** P<0.01.

We next overexpressed human MRP1 in ARPE-19 cells to study whether MRP1 overexpression would affect GSH and GSSG release. Real-time PCR and immunoblot analyses established the level of overexpression (Fig. 8A, B) in MRP1 transfected cells. GSH release was significantly (P<0.01) higher in MRP1 overexpressing than vector controls treated with H_2_O_2_ for 5 h (Fig. 8C). There was no significant change in LDH release in MRP1 overexpressing cells when compared with control cells indicating that GSH release was not due to toxicity (Fig. 8D). Intracellular GSH levels in MRP1 overexpressing cells (6.2±2.4 nmol/mg protein) were significantly (P<0.05) lower than vector control cells (9.9±1.7 nmol/mg protein).

We further examined the effect of H_2_O_2_ (150 µM) exposure for 5 h, 24 h, and 36 h in control and MRP1 overexpressing cells. The extent of cell death did not differ between control and MRP1 overexpressing cells at a shorter duration (5 h) of H_2_O_2_ treatment (Fig. 8D). However, at 24 h and 36 h of H_2_O_2_ treatment, a progressive increase in cell death was seen in control cells. Oxidant-induced cell toxicity in MRP1 overexpressing cells was significantly higher (P<0.05) than that seen in vector alone control cells (Fig. 8D). This finding was corroborated by levels of caspase 3 activation which progressively increased as the duration of H_2_O_2_ exposure increased (Fig. 8E). To explore the mechanism of cell death, we determined the GSH and GSSG levels in MRP1 overexpressed cells treated with H_2_O_2_ for 36 h. Cellular GSH levels were reduced by 32% in MRP1 overexpressed cells compared to vector control cells (Fig. 8F). H_2_O_2_ treatment further significantly (P<0.01) decreased cellular GSH levels by 25% and 62%, respectively in vector control cells and MRP1 overexpressed cells (Fig. 8F). However, GSSG levels were markedly lower in MRP1^+^ stressed as well as unstressed cells when compared to vector alone cells (Fig. 8G). With regard to efflux, MRP1 overexpressed cells effluxed significantly higher amounts of GSH vs vector controls with or without exposure to H_2_O_2_ (Fig. 8C, H). On the other hand, GSSG release was very low in MRP1 overexpressed cells under stressed as well as unstressed conditions (data not shown). These data show that MRP1 overexpression enhances RPE susceptibility to oxidant induced cell death due to low cellular GSH by increased GSH efflux.

## Discussion

RPE cells and retina from α-crystallin KO mice are highly susceptible to oxidant injury [23], [28]. Though multiple molecular mechanisms have been proposed to account for the function of crystallins in apoptosis [18], the role of GSH or thiols in this process has not received much attention. Depending on the severity of oxidant injury, cells undergo either GSH-dependent apoptosis or GSH-independent necrosis [31]. We have demonstrated that H_2_O_2_-induced cell death in α-crystallin KO RPE cells was due to apoptosis [23]. The dose of H_2_O_2_ used in the current study was previously shown by us to induce ROS production in RPE cells [32]. Here we show that apoptosis induced by H_2_O_2_ decreased significantly from about 30% in the control to 10% in the α-crystallin overexpressing cells. The protection was positively correlated with intracellular GSH and with mitochondrial GSH, supporting the notion that the modulation of ROS production was GSH-dependent in RPE cells. This is also consistent with earlier observations that small heat shock proteins were unable to protect against the oxidative insult generated by agents that interfere with GSH synthesis [22].

Mitochondrial GSH of RPE cells increased 2 fold with H_2_O_2_ treatment and by an increase in the cytosolic GSH. The increased cytosolic GSH triggers enhanced transport of GSH into mitochondria by activating specialized transport mechanisms [33]. In support of this finding, it has been demonstrated that in neuronal cells, hydrogen sulfide increases mitochondrial GSH [34]. Because apoptosis is closely linked to mitochondrial function, it can be argued that the H_2_O_2_-induced increase in mitochondrial GSH, rather than in cytosolic GSH in α-crystallin overexpressing cells may greatly contribute to cell protection. Retinal tissue fractions from α-crystallin KO mice showed decreased GSH levels, further supporting the link between GSH and α-crystallins in neuroprotection.

One of the mechanisms whereby cells maintain their redox status is by maintaining the GSH/GSSG ratio. The transporters involved in GSH release remain largely unknown, however, some studies describe involvement of MRPs in the transport of GSH and GSSG [15], [30], [35], [36], MRP1 is expressed in all mammalian cell types and is well characterized [9], [37]. Our data demonstrate that MRP1 is an effective transporter of GSH/GSSG in RPE cells. Cells treated with inhibitors of MRP decreased GSH release by about 50–70%. Similar findings have been reported in brain astrocytes that 60% of the GSH export is carried out by MRP1 [12]. In addition, selective knocking down of MRP1 caused a decrease in GSH release in unstressed and stressed conditions, providing direct evidence for the involvement of MRP1 in GSH-related cellular protection. We could not detect extracellular GSSG in MRP1 silenced RPE cells, a finding similar to that in astrocytes cultured from MRP1 KO mice [12]. Together, these data establish MRP1 as the major transporter of GSH and GSSG release in RPE.

Our studies further showed that MRP1 resides in the plasma membrane of non-polarized and polarized human RPE cells. MRP1 is localized to the basolateral membrane of epithelial cells in most tissues [38], [39]. Plasma membrane localization of MRP1 is critical for GSH transport. For example, it has been demonstrated that MRP1 is involved in GSH efflux in Jurkat cells where it is localized in the plasma membrane. In contrast, Raji cells lacked MRP1 at the plasma membrane and were unable to export GSH [35]. Levels of MRP1 were reported to increase after exposure to oxidative stress inducing agents [40]. We provide evidence that expression of MRP1 can be induced in cultured RPE treated with H_2_O_2_. Thus, the present study suggests that regulation of MRP1 in RPE cells under conditions of oxidative stress is redox sensitive and could help to maintain cellular homeostasis.

Intracellular GSH regulates the ability of cells to undergo apoptosis. Thus, experimentally increasing intracellular GSH decreases apoptosis while cells with lower GSH are more susceptible to apoptotic stimuli [41]. Intracellular GSH levels are regulated by three major ways during oxidant injury [42]: by inducing enzymatic synthesis of GSH via upregulation of GCLC, by the action of GR, which rapidly converts GSSG to GSH using NADPH as a substrate, and by cellular transport of GSH [42]. Our data indicate that the extracellular GSH transport mediated by MRP1 in response to oxidative injury may predispose RPE cells to caspase-mediated apoptosis given the known role of MRP1 in GSH and GSSG release [15]. Our study shows that GSSG levels were also increased in MRP1 silenced RPE cells and oxidative injury further increased GSSG by 4 fold. However, MRP1 silencing allows RPE cells to maintain their intracellular redox potential by upregulating GR activity which rapidly converts the toxic GSSG to GSH and may enhance cell survival. Similar findings were reported in human aortic endothelial cells where MRP1 inhibition prevented the decline in intracellular GSH, and reduced apoptosis caused by oscillatory shear by increasing GR activity [43]. Inhibition of MRP1 increased cellular GSH levels and reduced intracellular ROS and prevented angiotensin-induced apoptosis in endothelial progenitor cells [44]. In addition, *in vivo* studies show that the rate of apoptosis was significantly reduced in MRP1 KO mice and improved re-endothelialization after carotid artery injury [44]. Thus, multiple mechanisms may be operative in MRP1-inhibited cells that are more resistant to apoptosis.

On the other hand, we found that MRP1 overexpressing RPE cells release more GSH under unstressed and stressed conditions, further confirming the role of MRP1 as an effective GSH transporter [36], [45]. Because of the increased GSH release, steady state intracellular GSH levels are significantly lower in MRP1 overexpressing cells [36], [45], [46]. Our study demonstrated that under milder conditions of oxidative stress (5 h exposure to 150 µM H_2_O_2_) RPE cells remain viable and GSH release in MRP1 overexpressing cells was increased without affecting intracellular GSH levels, presumably because GSH biosynthesis was stimulated by a feedback mechanism [36]. However, prolonged treatment with H_2_O_2_ (36 h exposure to 150 µM H_2_O_2_) significantly increased the percentage of apoptotic cells and caspase activation in MRP1 overexpressing cells compared to control cells. It is well known that treatment with peroxides depletes GSH levels in RPE cells [47] leading to apoptosis. Thus, enhanced GSH release and depletion of intracellular GSH are important for the progression of apoptosis, and this phenomenon is applicable to MRP1 overexpressing cells with prolonged H_2_O_2_ exposure where the levels of cellular GSH is reduced by 62% and efflux increased by 1.8 fold. In support, similar results were reported in V79 Chinese hamster cells overexpressing MRP1 which did not show increased resistance to multiple stressors [45]. Similarly, treatment of MRP1 overexpressing BHK-21 cells with either verapamil or its derivative rapidly depleted intracellular GSH content with a strong decrease occurring during the first hour of treatment, followed by apoptosis [46]. The overexpression of MRP1 in HeLa cells while contributing to cell death by oxidative stress through enhanced GSH efflux also prevents intracellular GSSG accumulation [43], [48]. Thus the cell death observed in MRP1 overexpressing cells can be attributed to accumulation of ROS from GSH depletion [48]. However, in another study intracellular GSH levels were not depleted in MRP1-overexpressing HEK293 cells treated with staurosporine/Fas antibody despite increased GSH release [36]. These discrepant findings may be explained by differences in the duration of stress, different stressors tested, levels of MRP1 overexpression, and difference in cell lines or variable GSH levels maintained during experimentation among various studies.

While our studies address mainly the regulation and function of GSH as a MRP1 substrate, the patho-physiological significance of GSSG which is also transported by MRP1 cannot be overlooked given its cytotoxicity [14]. With this in mind, we also determined cellular levels and transport of GSSG in MRP1-silenced and MRP1 overexpressed RPE cells. As expected, cellular levels of GSH and GSSG significantly increased in MRP1-silenced RPE cells. However, the increased GSSG did not cause any adverse cytotoxicity since the expression of GR, the enzyme that converts GSSG to GSH, showed a significant increase in MRP1-silenced cells. Further, in control and MRP1 silenced RPE cells exposed to H_2_O_2_, the GR activity was upregulated elevating cellular GSH and thereby offering cellular protection (Fig. 7). Our observations are consistent with models of vascular abnormalities and hypertension in which MRP1 KO caused an increase in cellular GSH and GSSG levels while the increased activity of GR maintained the redox and protected cells from toxicity [14].

In summary, the present study describes the protective role of α-crystallin and interelation between GSH and MRP1 in RPE. RPE cells overexpressing α-crystallin are highly resistant to cell death due to higher intracellular GSH levels and the redox status is maintained by the efflux protein MRP1. Our results also show a compensatory upregulation of GR with H_2_O_2_ treatment as in human aortic endothelial cells [43]. On the other hand, MRP1-overexpressing cells exposed to oxidative stress are susceptible to apoptosis from decreased GSH levels caused by increased GSH efflux. Taken together, our results demonstrate a direct interaction among α-crystallin, GSH, and MRP1 in RPE cells and provide evidence that MRP1 regulates GSH homeostasis by different ways during oxidative stress. Enhancing the cellular defenses that protect the retina and RPE against oxidative stress has been a therapeutic objective aimed at reducing the progression of AMD. The evidence linking the protective role of α-crystallin and GSH and characterization of a transporter for GSH release offers new avenues for the use of these proteins in the therapy of ocular pathology.

## Materials and Methods

### Ethics statement

This study conforms to applicable regulatory guidelines at the University of Southern California, principles of human research subject protection in the Declaration of Helsinki and principles of animal research in the Association for Research in Vision and Ophthalmology Statement for the Use of Animals in Ophthalmic and Vision Research. The Institutional Review Board (IRB) of the University of Southern California approved our use of human RPE cells under protocol #HS-947005 (continuing review approved June 7, 2011). Human fetal eyes (16–18 week gestation) were obtained from Advanced Bioscience Resources Inc. (ABR, Alameda, CA) and written informed consent was obtained from all donors. The University of Southern California Institutional Animal Care committee approved our animal studies under protocol # 11135 (continuing review approved January 26, 2011).

### Cell Culture

ARPE-19 cells were obtained from American Type Culture Collection (ATCC, Manassas, VA). The protocol for generation of long-term polarized human fetal primary RPE cultures has been described in detail previously [49]. Human fetal eyes (16–18 week gestation) were obtained from Advanced Bioscience Resources Inc. (ABR, Alameda, CA). Isolation of RPE cells from α-crystallin KO and WT mice was carried out as described earlier [23].

### Construction of αA and αB-crystallin cDNAs

Full-length αA and αB-crystallin cDNAs were amplified from human fetal lens and fetal RPE, respectively, and cloned into a mammalian expression vector. Briefly, full-length α-crystallin cDNA (αA and αB crystallin) were amplified using the primer sequences (Table S1). The PCR products were digested with EcoR1 and Zho1, and then ligated into pcDNA 3.1 mammalian expression vector (Invitrogen, Carlsbad, USA) having a neomycin resistance gene for selection. Sequences were confirmed by DNA sequencing in the core facility of the Norris Cancer Center of the University of Southern California.

### Generation of stable cell lines

In order to ensure consistency in transfection studies, stable transfections were performed in ARPE-19 cells. Cells were transfected with the neomycin–resistant pcDNA vectors containing αA or αB crystallin inserts using FuGene 6 transfection reagent (Roche, IN). Cells were allowed to recover in DMEM/HAM's F12 with 10% FBS for 24 h and were sub-cultured in selection medium containing 500 µg/ml G418 sulfate (Sigma, St. Louis, MO). After 3 weeks, individual colonies were isolated, sub-cultured, expanded and examined for expression of αA and αB crystallin by immunoblot analysis with anti-αA and anti-αB crystallin antibodies (Novus Biologicals, Littleton, CO; Enzo Life Sciences, Farmingdale, NY).

### MRP1 overexpression

Generation of the human MRP1 cDNA cloned into the pcDNA 3.1 vector has been described [50]. ARPE-19 cells were transfected with the MRP1 pcDNA 3.1 vector and 48 h after transfection, mRNA and protein was isolated. Expression of MRP1 in the transfected cells was determined by real-time RT-PCR and by immunoblot analysis using a mouse monoclonal MRP1 antibody (Santa Cruz Biotech, Santa Cruz, CA). Cellular toxicity was determined by LDH assay [25].

### siRNA-mediated downregulation of MRP1 and αB crystallin

ARPE-19 cells at 50–60% confluence were transfected with pre-designed siRNA-MRP1, αB crystallin duplexes or scrambled siRNA (Dharmacon, Lafayette, CO) using HiPerFect transfection reagent (Qiagen, Valencia, CA). MRP1 mRNA and protein expression were analyzed by real-time RT-PCR and immunoblot analysis, respectively. αB crystallin protein expression was determined by immunoblot analysis.

### Detection of Apoptosis

αA and αB crystallin and empty vector clones grown on 4-well chamber slides were starved overnight in 1% FBS-containing medium and treated with 150 µM H_2_O_2_ for an additional 24 h. Cell death was assessed by Terminal deoxynucleotidyl transferase dUTP nick end labeling (TUNEL) following the manufacturer's protocol (Roche, IN). TUNEL positive cells were counted and quantified as described [21], [26].

### GSH and GSSG Analysis

Total cellular glutathione content in RPE/choroid complex and neural retina was measured following the manufacturer's protocol. Mitochondria and cytosol were isolated using a Mitochondria/Cytosol fractionation kit [23]. GSH and GSSG levels were measured with a commercially available kit (Oxford Biomedical Research, Oxford, MI). Total GSH levels were expressed either as µmol/ml or nmol/mg total protein and were normalized to % of controls.

### GSH and GSSG Efflux from RPE cells

Control ARPE-19 cells as well as cells from α-crystallin overexpressing, MRP1 overexpressing, and MRP1 siRNA treated groups were treated with H_2_O_2_ in serum-free culture medium for 5, 24 or 36 h. After the experimental period, medium was collected, centrifuged to remove dead cells and debris and GSH and/or GSSG release was determined in the cell-free medium. Total protein was isolated from the cells, quantified (Bio-Rad Laboratories, Hercules, CA) and intracellular GSH or GSSG content was measured. GSH release was expressed as nmol/ml per unit time.

### Immunoblot analysis

Cells were harvested after the specified treatment period and protein was extracted from the cells or posterior eye cups [28]. Equal amounts of protein (25–75 µg) were resolved on 15 or 4–15% Tris-HCl polyacrylamide gels as described previously [5], [26]. Membranes were probed with rabbit polyclonal glutamate-cysteine ligase, catalytic subunit (GCLC) (1∶1000, Cayman, Ann Arbor, MI), polyclonal glutamate-cysteine ligase, modifier subunit (GCLM) anti-MRP1 (1∶500), anti-glutathione reductase (1∶500, Santa Cruz Biotech, Santa Cruz, CA), anti-αA crystallin (1∶1000), anti-αB crystallin (1∶1000, Enzo Life Sciences), overnight at 4°C. After incubation with the corresponding secondary antibodies, signals were detected using an enhanced chemiluminescence system, membranes reprobed for GAPDH or β-actin.

### Quantitative real-time PCR

Total RNA was isolated from ARPE-19 cells or mouse posterior eye cups (neural retina, RPE and choroid) using TRIzol reagent (Invitrogen, CA), and RNA quantified. Reverse transcription and real-time PCR was performed as described earlier [26], [32]. The sequences of primers used are presented in Table S1. Relative multiples of change in mRNA expression was determined by calculating 2^−ΔΔCT^. Results are reported as mean difference in relative multiples of change in mRNA expression ± SEM.

### Immunofluorescence cell staining

Cells were grown on 4-well chamber slides or human fetal RPE monolayers on transwell filters were processed [25], [51]. After incubation with primary antibody (anti-MRP1 1∶50), slides were incubated with fluorescein -conjugated secondary antibody (Vector Lab, CA) and were examined using a laser scanning confocal microscope (LSM510, Zeiss, NY).

### Biotinylation

RPE cells (2×10^6^ per flask) at 90% confluence were used for biotinylation as suggested by the manufacturer (Thermo Scientific, Rockford, IL). Briefly, cells were incubated with 10 ml biotin solution on a shaker for 30 min at 4°C and the cells were gently scraped and collected by centrifugation. The cells were sonicated and incubated on ice for 30 min with vortexing in between every 5 min. The samples were centrifuged and the supernatant was added to the microcentrifuge spin column. The column was subjected to low speed centrifugation, and finally 300 µl of sample buffer was added to the column and incubated 1 hr at room temperature. The membrane fraction was collected by centrifugation and was subjected to immunoblot analysis.

### Data Analysis

Data were analyzed with InStat (GraphPad Software, San Diego, CA). ANOVA and Tukey post hoc test were used to assess the differences between groups. P<0.05 was considered to be statistically significant.

## Supporting Information

Figure S1 **Expression of redox family members in αA crystallin KO and WT mice.** Changes in redoxin mRNA (A) and protein (B) in WT and αA crystallin KO retina. mRNA and protein were extracted from the posterior eye cup. (A) Real-time PCR was used to amplify the mRNA levels. Data are normalized to L32 and presented as relative fold difference over control (WT). (B) 25–50 µg total protein was loaded for Western blot analysis and probed with rabbit Trx1, goat Trx2 and rabbit Grx1. GAPDH was used as a loading control. All four redox proteins showed a significant decrease in expression when compared to corresponding age-matched wild type. Trx1- Thioredoxin 1, Trx2- Thioredoxin 2, Grx1- Glutaredoxin 1, Grx2- Glutaredoxin 2. * P<0.05, ** P<0.01.
(TIF)

Figure S2 **Expression of MRP family members in RPE cells.** A. RT-PCR showing the expression of isoforms of MRPs in RPE cells. Agarose gel separation of the amplification products showed cDNA fragments of the expected size for MRP1, MRP2, MRP3, MRP4, MRP5, MRP6 and MRP7. See Suppl.Table 1 for the primers used. B. Real time–PCR showing the relative abundance of MRPs in RPE cells. Data presented are normalized with GAPDH as housekeeping gene and MRP1 as 1. MRP1 showed the highest abundance, followed by MRP5 and MRP7. * P<0.05, ** P<0.01.
(TIF)

Table S1 **PCR primers utilized in this study.**
(DOC)
