# Supplementary figures and images for: Mechanism of RPE Cell Death in α-Crystallin Deficient Mice: A Novel and Critical Role for MRP1-Mediated GSH Efflux
Source: PLoS One. 2012 Mar 19;7(3):e33420. doi: 10.1371/journal.pone.0033420 (PMC3307734; doi:10.1371/journal.pone.0033420)

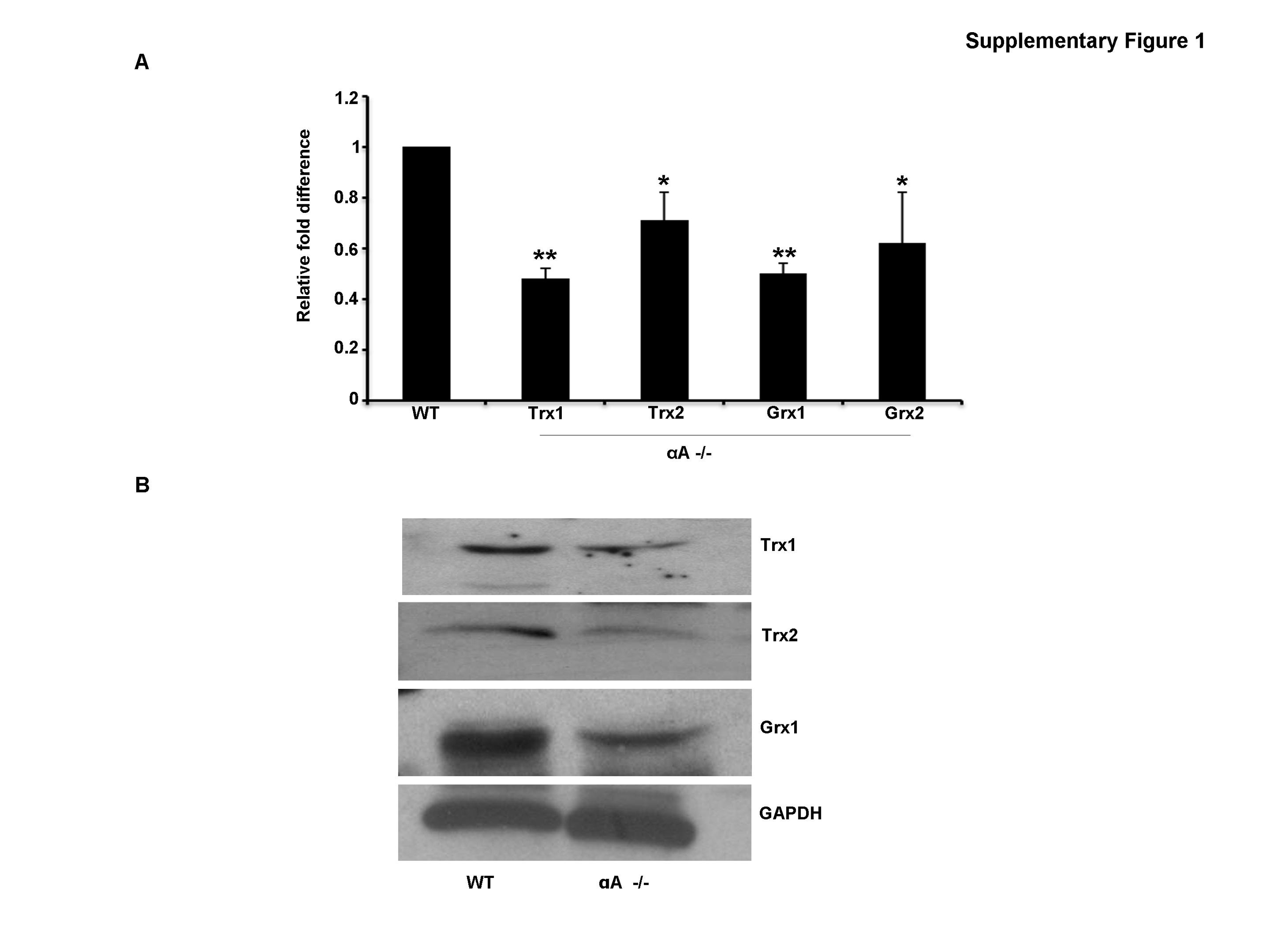

Supplement: Figure S1 — Expression of redox family members in αA crystallin KO and WT mice. Changes in redoxin mRNA (A) and protein (B) in WT and αA crystallin KO retina. mRNA and protein were extracted from the posterior eye cup. (A) Real-time PCR was used to amplify the mRNA levels. Data are normalized to L32 and presented as relative fold difference over control (WT). (B) 25–50 µg total protein was loaded for Western blot analysis and probed with rabbit Trx1, goat Trx2 and rabbit Grx1. GAPDH was used as a loading control. All four redox proteins showed a significant decrease in expression when compared to corresponding age-matched wild type. Trx1- Thioredoxin 1, Trx2- Thioredoxin 2, Grx1- Glutaredoxin 1, Grx2- Glutaredoxin 2. * P<0.05, ** P<0.01. (TIF) [file pone.0033420.s001.tif]

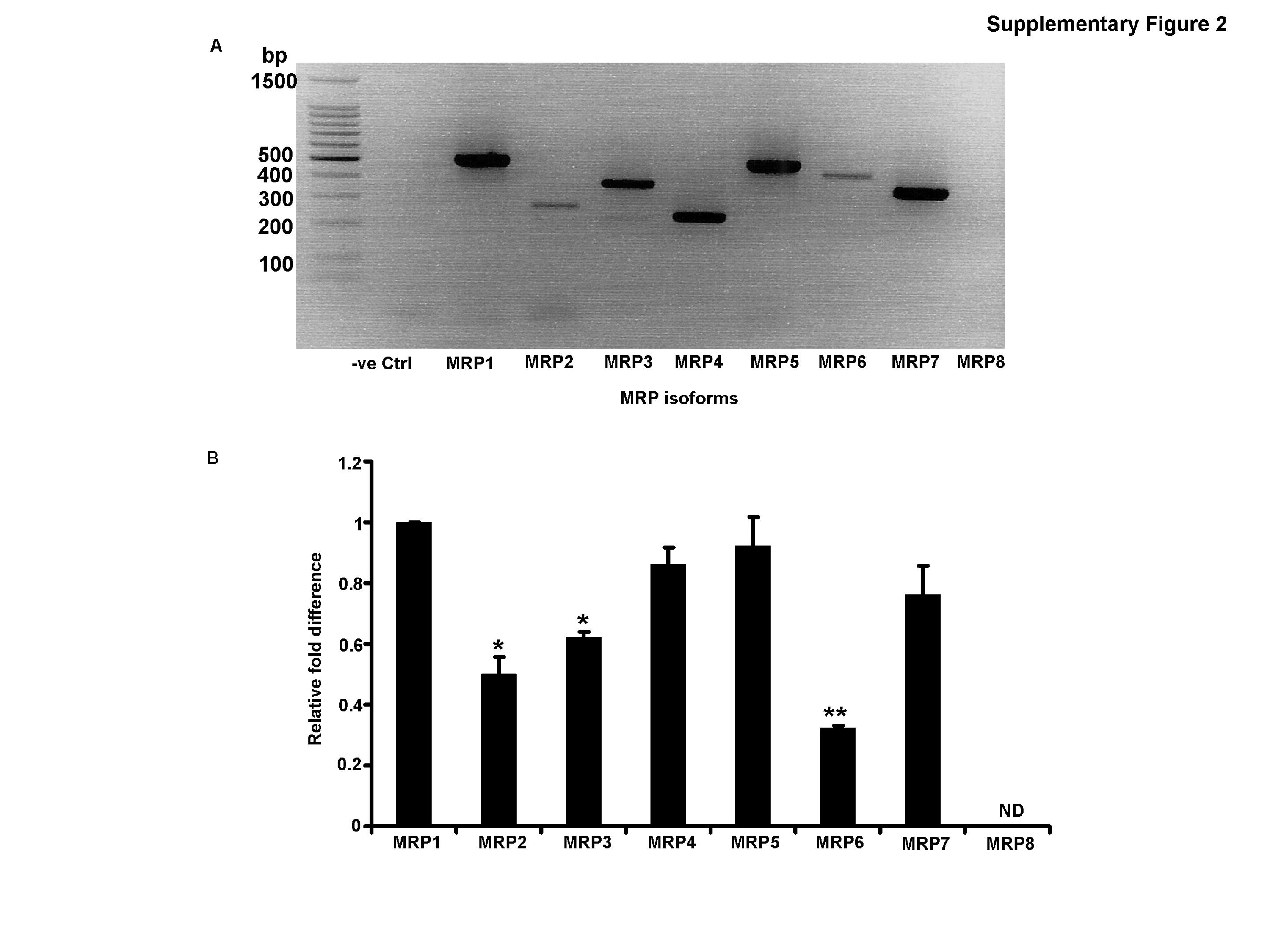

Supplement: Figure S2 — Expression of MRP family members in RPE cells. A. RT-PCR showing the expression of isoforms of MRPs in RPE cells. Agarose gel separation of the amplification products showed cDNA fragments of the expected size for MRP1, MRP2, MRP3, MRP4, MRP5, MRP6 and MRP7. See Suppl.Table 1 for the primers used. B. Real time–PCR showing the relative abundance of MRPs in RPE cells. Data presented are normalized with GAPDH as housekeeping gene and MRP1 as 1. MRP1 showed the highest abundance, followed by MRP5 and MRP7. * P<0.05, ** P<0.01. (TIF) [file pone.0033420.s002.tif]
