# Supplementary material for: Mechanism of RPE Cell Death in α-Crystallin Deficient Mice: A Novel and Critical Role for MRP1-Mediated GSH Efflux
Source: PLoS One. 2012 Mar 19;7(3):e33420. doi: 10.1371/journal.pone.0033420 (PMC3307734; doi:10.1371/journal.pone.0033420)
Supplement: Table S1 — PCR primers utilized in this study. (DOC) [file pone.0033420.s003.doc]

**Suppl. Table 1. Sequences of primers used in the study**

| **Human Primer Sequences** | |
| --- | --- |
| αA crystallin- F | CCACCATGGACATCGCCATCC |
| αA crystallin- R | GGGCATCTATTTCTTGGGGGCTGC |
| αB crystallin- F | GCTGAACATGGACGTGACCAT |
| αB crystallin- R | AATGCCTGCTTAGGACGAGGG. |
| MRP1-F | AGGTCAAGCTTTCCGTGTACTG |
| MRP1-R | GGACTTTCGTGTGCTCCTGA |
| MRP2-F | GCAGCGATTTCTGAAACACA |
| MRP2-R | CAACAGCCACAATGTTGGTC |
| MRP3-F | TCACCTATGTGGTCCAGAAG |
| MRP3-R | CTAAAGCAGCATAGACGCCC |
| MRP4-F | CCAVTGAAGATCTTCCTGG |
| MRP4-R | GGTG1TCAATCTGTGTGC |
| MRP5-F | ACCCGTTGTTGCCATCTTAG |
| MRP5-R | TCTGTCAACAGCCACTGAGG |
| MRP6-F | TCAGAAGCCCAGACAGAGGT |
| MRP6-R | CGGTTTAGCAGGTGACCAAT |
| MRP7-F | GAACGGCTGCTTAACTTTGC |
| MRP7-R | AGGTATACACAGGCCGCATC |
| MRP8-F | GCCAAAGGTAGAAAGTCAGGCT |
| MRP8-R | CTGAAGATCGTTAAGAAGACGATCA |
| GR-F | AACAACATCCCAACTGTGGTC |
| GR-R | CCATATTTATGAATGGCTTCATCTT |
| GCLC-F | ATGGAGGTGCAATTAACAGAC |
| GCLC-R | ACTGCATTGCCACCTTTGCA |
| GCLM-F | GCTGTATCAGTGGGCACAG |
| GCLM-R | CGCTTGAATGTCAGGAATGC |
| GAPDH-F | CGACCACTTTGTCAAGCTCA |
| GAPDH-R | GGTGGTCCAGGGGTCTTACT |
| **Mouse primer Sequences used** | |
| TRX1-F | ATGGTGAAGCTGATCGAGA |
| TRX1-R | CAGTAATAGAGGCTTCAAGC |
| TRX2-F | GGACCGCGGCTAGAGAAGAT |
| TRX2-R | GCTGGTCCTCGTCCTTGATC |
| GRX1-F | CAACACCAGTGCGATTCAAGA |
| GRX1-R | GCAGAGCTCCAATCTGCTTCA |
| GRX2-F | TGGAATATGGCAACCAGTTTCA |
| GRX2-R | GGCGACTATCCACATCATTCAA |
| GCLC-F | GATTCGGGATGGGCAACT |
| GCLC-R | AAAGGTATCTTGCCTCAGATATGC |
| GCLM-F | TGGAGCAGCTGTATCAGTGG |
| GCLM-R | CAAAGGCAGTCAAATCTGGTG |
| L32- F | TGGTTTTCTTGTTGCTCCCATA |
| L32-R | GGGTGCGGAGAAGGTTCAA |
